# Lung Ultrasound in Adults and Children with COVID-19: From First Discoveries to Recent Advances

**DOI:** 10.3390/jcm11154340

**Published:** 2022-07-26

**Authors:** Danilo Buonsenso, Luigi Vetrugno

**Affiliations:** 1 Department of Woman and Child Health and Public Health, Fondazione Policlinico Universitario A. Gemelli IRCCS, 00120 Rome, Italy; 2 Centro di Salute Globale, Università Cattolica del Sacro Cuore, 00120 Rome, Italy; 3 Department of Medical, Oral and Biotechnological Sciences, University of Chieti-Pescara, 66100 Chieti, Italy

During this pandemic, the lung ultrasound (LUS) imaging modality has shown promising results as a diagnostic, prognostic and monitoring tool for COVID-19 patients [1]. Clinicians experienced in LUS before the pandemic have rapidly incorporated LUS into the clinical armamentarium for the evaluation of non-critically and critically ill COVID-19 patients as a fast-to-use, repeatable, bedside chest imaging modality [2,3]. This editorial aims to summarize how LUS for COVID-19 has evolved during the pandemic, and the future challenges clinicians will face to improve the use of this tool (Figure 1).

In the largest international multicenter observational study, involving 20 US and European hospitals with patients suspected of having COVID-19, LUS patterns of probability integrated with patients’ characteristics allowed the diagnosis of COVID-19 pneumonia at the bedside with high accuracy, according to the different clinical phenotypes [4].

Since then, national and international scientific societies worldwide have officially acknowledged the role of LUS in COVID-19 patients [5,6,7,8]. A specific survey about LUS, named ITALUS, with 807 respondents, showed that LUS was extensively used during the first wave of the COVID-19 pandemic by anesthesiologists and intensive care physicians in Italy. However, 76.7% of this sample did not undertake any LUS certification [9].

However, three years and several coronavirus waves later, early recommendations about the use of LUS in non-critical and critically ill COVID-19 patients can be questioned, and must be updated as the perceived clinical impact on decision making becomes more precise.

*First*, LUS has shown higher accuracy than chest X-ray (CXR) in the first stage of the disease, and a more reasonable correlation with thoracic high-resolution computed tomography (HRCT) scan imaging modalities after hospital admission, as shown in a previous multicentric observational study [10,11].

*Second*, compared with chest X-rays, a more reasonable diagnostic impact has also been demonstrated, especially in vulnerable populations such as pediatric patients and pregnant women, where ultrasound can be used as a point-of-care repeatability image modality, without radiation exposure, at a low cost [12]. High-resolution computed tomography imaging may be useful in moderate to severe disease where patients demonstrate worsening gas exchange or a lack of improvement in respiratory status with time, and this method remains the gold-standard diagnostic image modality, especially in cases of suspected pulmonary embolism. However, it should be remembered that although the HRCT scan and ultrasound are sensitive, they are not specific for the diagnosis of COVID-19 [11].

*Third*, a single-center study of Lichter et al. in 120 consecutive adult patients who underwent complete LUS within 24 h of ICU admission during the first COVID-19 wave showed that the baseline LUS score correlates with the eventual need for invasive mechanical ventilation and is a strong predictor of mortality [13]. An French multicentric ancillary retrospective study in an ICU did not confirm this finding with regard to LUS performance for predicting the 28-day mortality rate in intubated SARS-CoV-2 patients, instead finding that the LUS score was better for non-intubated patients at admission [14]. Similar findings have also been reported in Italian ICU studies [15,16]. In agreement with these French and Italian studies, Baciarello et al. [17] found the clinical usefulness of the LUS score during the COVID-19 outbreak in the setting of medical wards where LUS was useful in identifying and monitoring those patients with persistent PaO_2_/FiO_2_ ≤ 200 mmHg found that LUS was significantly higher in patients who were eventually transferred to the ICU for intubation and invasive mechanical ventilation (IVM) or who died. Therefore, the LUS score presents a better predictive value for non-intubated patients at admission.

*Fourth*, in ICU daily clinical practice, LUS currently represents the first-line diagnostic tool for ICU physicians to rule out possible acute complications such as pneumothorax, supra-infections and large pleural effusion, reducing the need for CT scan and the consequent potential risk of aerosol dispersion of the virus during transport to the radiological ward, saving the time required for the diagnostic image modality and reducing strain on the limited resources available. The 12-zone scanning for LUS protocol is the most useful for diagnosing and monitoring COVID-19 pneumonia [1,6]. Meroi et al. calculated the LUS score in 25 patients admitted to the COVID-19 ICU and the time needed to perform the exam [18]. They reported a median time of 4.2 min (IQR 3.6–4.5) despite the personal protective equipment limiting mobility, achieving a median time that was roughly half that found by Rouby et al. [19]. The semi-continuous daily evaluation adds value to the clinical context considering that COVID-19 patients who require mechanical ventilation for ARDS have a high risk (> 50%) of developing ventilator-associated pneumonia (VAP), most commonly because of Gram-negative bacteria, and that LUS can help to differentiate between bacterial colonization versus superinfection [20].

*Fifth*, Vetrugno et al. [21] found that semi-continuous assessment with LUS score associated with the clinical context could help to understand the patient trajectory, and described four phenotypes:

(a) Phenotype 1: patients with clinical improvement independent from the LUS evolution;
(b) Phenotype 2: patients who presented a moderate improvement in their ultrasound imaging;
(c) Phenotype 3: patients who responded very clearly, with a significant reduction in pulmonary involvement and LUS score;
(d) Phenotype 4: patients who, while improving their clinical conditions, did not show an evident improvement from an ultrasound point of view, or presented an apparent worsening in LUS.

This is in agreement with the first studies indicating that LUS is a good tool to identify and monitor pneumonia by the assignment of increasing scores. However, new studies showed that the lung ultrasound picture could be confused by other factors such as cardiac disease, patient fluid management and diaphragmatic function, factors not included in the LUS score [22,23]. Therefore, lung ultrasound skills require more diffused training and guidance to ensure the necessary support to non-critical and critically ill physicians.

The World Alliance Societies of Echocardiography (WASE-COVID) studied 870 patients with acute COVID-19 infection from 13 medical centers in four world regions (Asia, Europe, United States, and Latin America) having undergone transthoracic echocardiograms [22]. The authors found that LV dysfunction was observed in approximately 20% and RV dysfunction in approximately 30% of patients with acute SARS-CoV-2 infection and that age at presentation, previous lung disease, LDH, LVLS, and RVFWS were independently associated with in-hospital mortality. These new findings support the interest in an integrated evaluation of the lungs and cardiac ultrasound function to evaluate the patient’s prognosis, considering that respiratory insufficiency can be a multifactorial decision-making process [22,23,24]. However, the routine use of echocardiography among low-risk patients is not recommended.

Recently, Dell’Acquila et al. questioned the fact that the LUS score does not include parameters such as inferior vena cava (IVC) diameter, the index of collapsibility or distensibility, diaphragmatic excursions and the evaluation of pleural and pericardial effusions [25]. They proposed a new scoring system, termed “integrated00” lung ultrasound score (i-LUS), which incorporates the inferior vena cava (IVC), diaphragmatic excursions and pleural and pericardial effusions. They found that i-LUS could be a helpful clinical tool for early decision making in patients with COVID-19 pneumonia [25].

***Specificities of LUS in Children with COVID-19***

Pediatricians started implementing LUS approximately ten years before the beginning of the pandemic [26], with some centers already using it as a standardized clinical tool and with a formal report form [27]. Therefore, it was not unexpected that LUS was evaluated in pediatric COVID-19.

The first pediatric descriptions confirmed that pediatric COVID-19 was also characterized by mostly peripheral lung localizations, including cases in asymptomatic children [28], therefore making LUS a feasible option given its recognized role in characterizing the peripheral lung. LUS was used both in China and Italy as soon the first pediatric cases were detected, including cases in newborns [29,30].

Achievements in pediatric practice include:

- Confirmation that LUS was able to detect peripheral lung consolidations in children, independent from disease severity [28];
- The optimum correlation between LUS and CT findings, making LUS a safe option to assess children with COVID-19 and spare them from unnecessary radiation, at least in asymptomatic, mild and moderate disease [31];
- Equal or better sensitivity than chest X-ray in detecting lung involvement during SARS-CoV-2 infection [32];
- Usefulness in monitoring lung involvement during follow-up, shown in small studies and supported by previous experience from other viral pediatric respiratory infections [33];
- Application in children with Multisystem Inflammatory Syndrome, one of the most severe post-infective complications of SARS-CoV-2 infection [34,35]. This finding is in agreement with previous pediatric studies that showed how lung ultrasound can detect cardiopulmonary interactions during acute systemic diseases [36]. LUS has also been successfully used in MIS-C. Specifically, a team from Rome described the first findings of vertical artifacts and simple pleural effusions, and a team from Northern Italy detected a cutoff of severity, predicting the need for intensive care admission, as well as inotropic or respiratory support [34,35]. Importantly, as MIS-C is a systemic condition, a wider application of point-of-care ultrasound that also assesses free abdominal fluids, the thickening of intestinal walls, and ventricular contraction would allow a better recognition and characterization of a child with suspected MIS-C, as described by two independent teams [37,38].

Importantly, LUS has failed to be able to specifically distinguish SARS-CoV-2 pneumonia from other viral pneumonias, which have similar LUS semeiotics and overlapping physio-pathologic events. In this regard, LUS may be a promising tool to support the characterization of the main etiological category of lower respiratory tract infection (viral vs. bacterial vs. atypical), but it does not seem realistic to believe that LUS can also provide specific patterns for each etiological agent [39,40].

Differently from adult practice, a number of specific characteristics of pediatric COVID-19 make the routine use of LUS in children with confirmed SARS-CoV-2 infection easier:

- The possibility of using linear high-frequency probes in the majority of cases;
- Rare chronic respiratory comorbidities that make the interpretation of LUS findings more difficult;
- A well-established lower risk of developing severe COVID-19, including thromboembolic events, which may be more difficult to recognize with the exclusive use of LUS.

Given these considerations, the role of LUS in pediatric COVID-19 has not significantly changed and still has the potential to be used as a screening tool to diagnose pneumonia in acute infection and monitor lung involvement during follow-up, sparing patients from unnecessary radiation.

A current gap in the literature is represented by the lack of a cutoff score for peripheral lung involvement, which might be able to predict those children that would most benefit from closer observation given the possible risk of deterioration, as has been established in children with bronchiolitis. However, this goal seems difficult to achieve, since severe and critical COVID-19 is extremely rare in children and, therefore, it may be difficult to include a sufficient number of children to cover the full spectrum of severity. In fact, the studies that have tried to prove a prognostic role have only included very small numbers of patients, and we therefore cannot currently support the use of LUS to predict which children will develop more severe disease. Similarly, as new evidence is coming to light on the impact of long COVID in children, it may be worth investigating whether children with long COVID experience persisting lung pathology/inflammation compared to those that have fully recovered. However, a solid study would also need pre-COVID LUS findings for each patient, making such a study design very difficult to make feasible. A control group of completely healthy children would be necessary, since the LUS findings of healthy children from different age groups have never been documented. In healthy neonates and infants, for example, we recently demonstrated that vertical artifacts are common and consolidations are absent, suggesting that vertical artifacts may represent an immature lung periphery and, therefore, such evidence in young children needs to be interpreted according to clinical information. As the normal lung in older children has never been characterized by LUS, a study aimed at investigating LUS in long COVID would also need to assess the normal lung in this group.

***Conclusions***

The role of LUS in adults and children with COVID-19 is now more consolidated, although there is increasing evidence that the optimal use of LUS may be in the context of a wider point-of-care ultrasound approach for patients with COVID-19, aimed at addressing at least the lung, the heart and major explorable vessels, with the addition of searching for free abdominal fluid and bowel thickening in cases of suspected MIS-C. Importantly, future prospective studies should consider that the “first” virus variant was suitable for lung ultrasound detection, mainly located in the bases of the lungs and the peripheral regions. However, this will require reassessment as different COVID-19 variants emerge. Finally, we encourage more attention to be paid to cleaning ultrasound equipment before every exam.

## Figures and Tables

**Figure 1:**
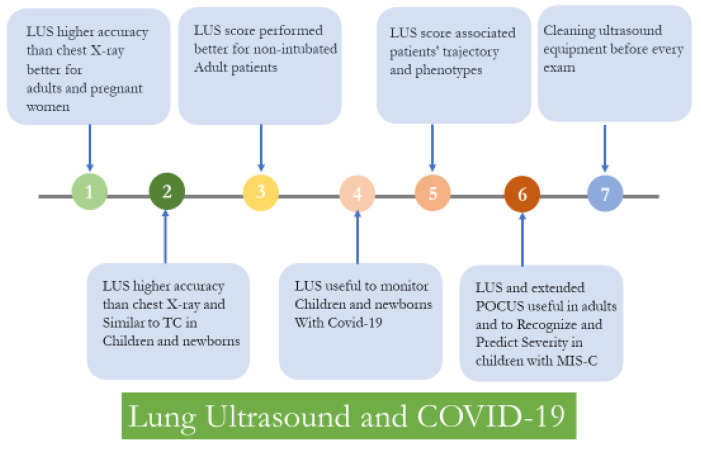
Main steps in the implementation of lung ultrasound in patients with COVID-19.
